# Rivaroxaban-induced spontaneous hemothorax: a rare case report and literature review

**DOI:** 10.3389/fmed.2025.1641092

**Published:** 2025-07-29

**Authors:** Xiaolong Li, Shuhao Xu, Chunfang Zeng, Ding Han, Xin Wang

**Affiliations:** ^1^Pulmonary and Critical Care Medicine, Deyang People’s Hospital, Deyang, China; ^**2**^Department of Stomatology, Deyang People's Hospital, Deyang, China

**Keywords:** rivaroxaban, fluconazole, spontaneous hemothorax, drug–drug interaction, case report

## Abstract

**Introduction:**

Rivaroxaban, a novel oral anticoagulant, is widely used in patients with non-valvular atrial fibrillation. Bleeding events during rivaroxaban therapy most commonly include gastrointestinal bleeding and intracranial hemorrhage, while spontaneous hemothorax is exceedingly rare. We report a case of spontaneous hemothorax occurring during rivaroxaban administration.

**Methods:**

We report the case of a patient presenting primarily with dyspnea. The patient had been on long-term oral rivaroxaban for atrial fibrillation. Physical examination upon admission revealed diminished breath sounds in the left lung. Computed tomography imaging demonstrated a large left-sided pleural effusion with adjacent pulmonary atelectasis. Laboratory tests indicated significant coagulation abnormalities. After admission, the patient underwent therapeutic thoracentesis and chest tube placement, with bloody pleural fluid observed in the drainage. Due to the marked coagulation abnormalities on admission, the patient received fresh frozen plasma transfusion. Further history-taking revealed recent use of oral fluconazole. A review of the literature suggested that the spontaneous hemothorax might be associated with the concurrent use of rivaroxaban and fluconazole.

**Results:**

Following treatment, a follow-up computed tomography scan 3 months later showed no evidence of recurrent hemothorax.

**Discussion:**

Concomitant administration of rivaroxaban and fluconazole significantly increases the risk of bleeding events, necessitating clinician vigilance regarding potential drug–drug interactions when formulating therapeutic regimens.

## Introduction

Rivaroxaban, a novel oral anticoagulant, is widely used in patients with non-valvular atrial fibrillation (AF). Bleeding events associated with rivaroxaban in this population have been frequently reported, with gastrointestinal bleeding and intracranial hemorrhage being the most common (1, 2). Additionally, some patients may experience conjunctival bleeding, genitourinary bleeding, gingival bleeding, subcutaneous hemorrhage, hemoptysis, and other bleeding manifestations. However, spontaneous hemothorax represents an exceedingly rare complication. This article reports a case of spontaneous hemothorax occurring in a patient during rivaroxaban therapy.

### Case presentation

A 77-year-old man was admitted to our hospital due to shortness of breath for 1 month. The elderly male patient presented with dyspnea for approximately 1 month, which was particularly noticeable during physical activity. He did not exhibit symptoms such as fever, night sweats, chest pain, hemoptysis, orthopnea, or altered consciousness.

**Figure 1:**
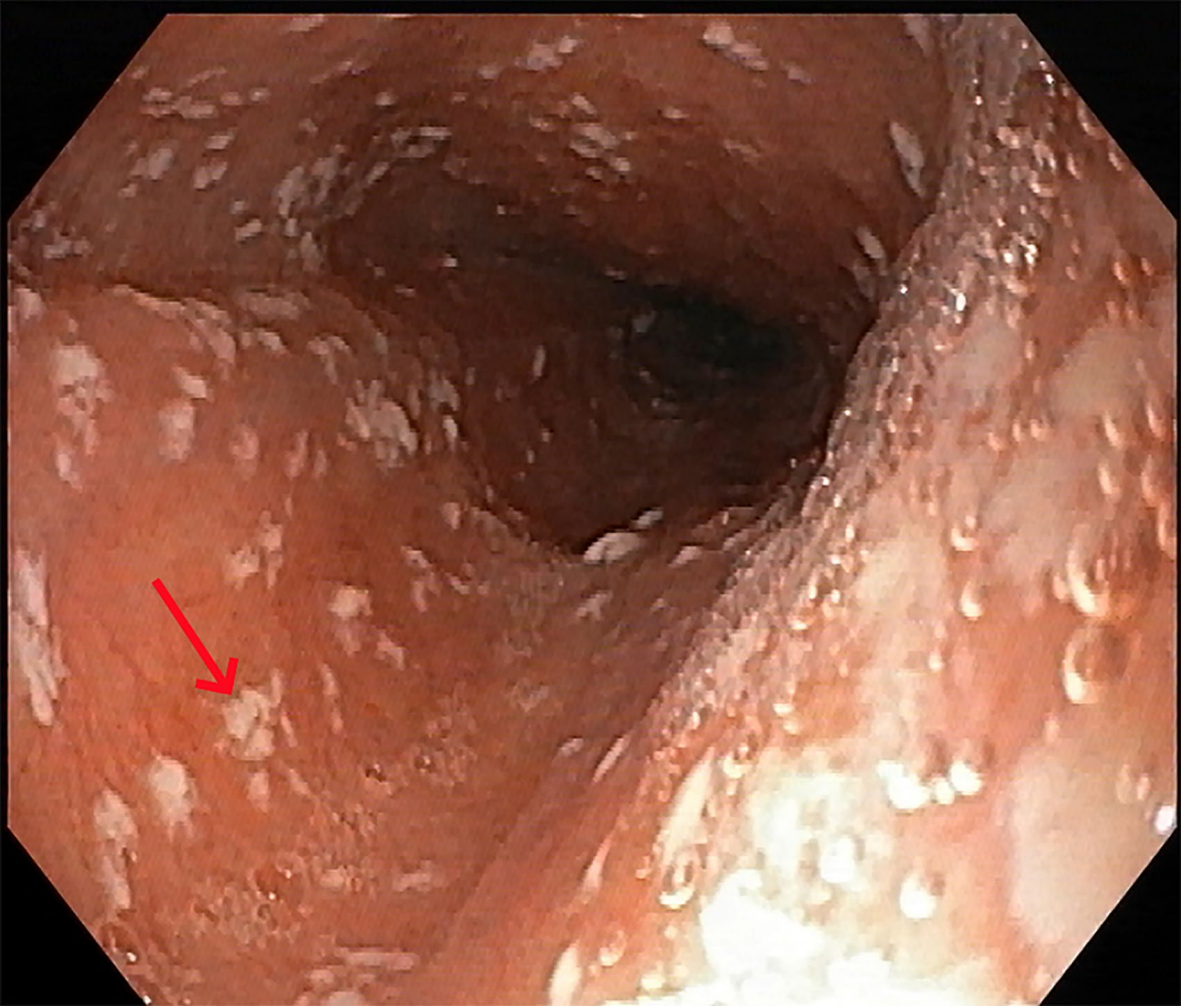
Upper gastrointestinal endoscopy findings. Grayish-white plaques were observed on the esophageal mucosal surface, with accompanying mucosal congestion and edema (red arrow).

The patient has a history of non-valvular AF with bradycardia, for which a cardiac pacemaker was implanted 2 years ago. He has been on long-term oral rivaroxaban 15 mg once daily. One and a half months previously, the patient presented to the gastroenterology department with pain during swallowing. An upper gastrointestinal endoscopy was performed, revealing esophageal candidiasis (Figure 1). The patient was subsequently treated with oral fluconazole for 2 weeks, with a loading dose of 400 mg on the first day followed by a maintenance dose of 200 mg once daily.

The patient has a 30-year history of smoking and has abstained from smoking for 2 years. Additionally, he has a 30-year history of alcohol consumption and has been abstinent for over 5 years. Family history is negative for infectious diseases, diabetes, pulmonary tumors, anxiety, depression, or hematologic disorders. No relevant genetic history is reported.

Upon admission, the patient’s respiratory rate was 18 breaths per minute, heart rate was 103 beats per minute, blood pressure was 109/76 mmHg, and peripheral oxygen saturation was 91%. Physical examination revealed diminished breath sounds on the left lung, with no other significant abnormalities noted.

Laboratory tests revealed the following results: white blood cell count 7.94 × 10^9^/L, neutrophil count 6.09 × 10^9^/L, lymphocyte count 0.89 × 10^9^/L, eosinophil count 0.09 × 10^9^/L, monocyte count 0.85 × 10^9^/L, basophil count 0.3 × 10^9^/L, red blood cell count 4.05 × 10^9^/L, hemoglobin 117 g/L, platelet count 354 × 10^9^/L, and high-sensitivity C-reactive protein 10.74 mg/L. Brain natriuretic peptide was 174.2 pg/mL, and liver and kidney function tests were within normal limits. Procalcitonin was 0.012 ng/mL, prothrombin time (PT) 29.70 s, international normalized ratio (INR) 2.73, activated partial thromboplastin time (APTT) 52.00 s, fibrinogen (FBG) 5.53 g/L, thrombin time (TT) 19.80 s, fibrinogen degradation products (FDP) 8.36 mg/L and D-dimer (D-D) 3.45 mg/L FEU (Table 1). Tumor markers, including carcinoembryonic antigen, neuron-specific enolase, squamous cell carcinoma antigen, and cytokeratin 19 fragment, were all within normal ranges. The purified protein derivative test was negative. Laboratory tests indicated normal liver and kidney functions.

**Table 1:** Coagulation parameters at admission and post-treatment.

Test item	Results (at admission)	Results (after treatment)	Reference value
PT (s)	29.70	14.20	9.70–14.20
INR	2.73	1.25	0.86–1.26
APTT (s)	52.00	29.50	24.80–33.80
FBG (g/L)	5.53	4.44	2.00–4.00
TT (s)	19.80	19.80	14.00–21.00
FDP (mg/L)	8.36	4.06	0.00–5.00
D-D (mg/L FEU)	3.45	0.95	0.08–0.55

**Figure 2:**
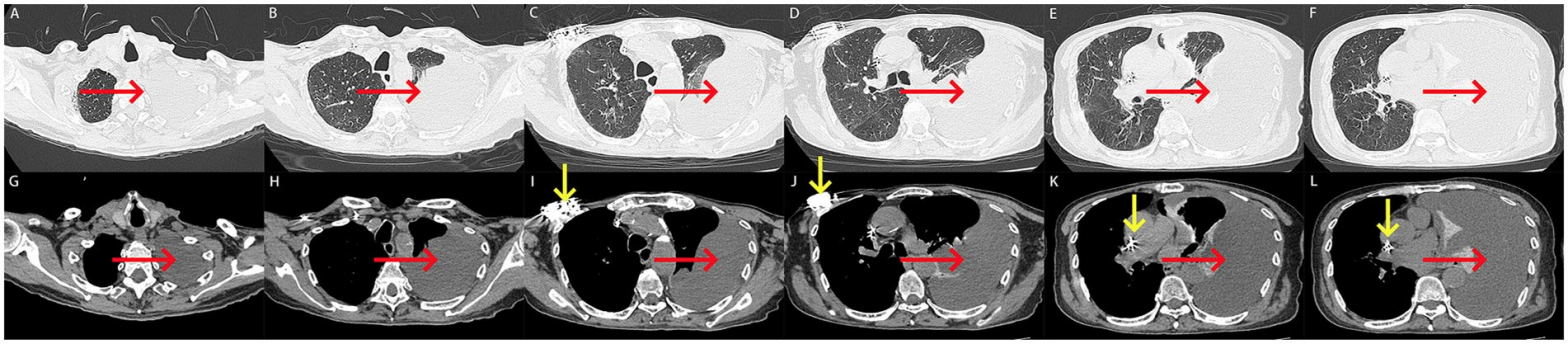
**(A–L)** Computed tomography scan on admission. **(A–L)** A large left-sided pleural effusion with adjacent pulmonary atelectasis was revealed on computed tomography (red arrow). A cardiac pacemaker was seen on computed tomography (yellow arrow).

Computed tomography (CT) performed on August 5, 2024, revealed a large left-sided pleural effusion with adjacent pulmonary atelectasis and the presence of a cardiac pacemaker (Figures 2A–L).

Based on the patient’s symptoms, laboratory findings, and CT imaging, the diagnosis was established as a large left-sided pleural effusion, mild anemia, and status post cardiac pacemaker implantation. The large left-sided pleural effusion required differentiation from other potential etiologies, such as tuberculous pleuritis and malignant pleural effusions.

Upon admission, the patient received oxygen therapy, therapeutic thoracentesis, and placement of a chest drain. The pleural fluid drained through the chest tube appeared bloody and was non-coagulable. The laboratory analysis demonstrates a hematocrit level of 28.8% in pleural effusion compared with 36.4% in peripheral blood, which collectively meet the diagnostic criteria for hemothorax. Laboratory analysis of the pleural fluid was performed (Table 2). Cytological examination of pleural effusion indicated that no tumor cells were observed in the pleural effusion. Given the absence of recent trauma or surgery, the patient was diagnosed with spontaneous hemothorax. Considering the patient’s ongoing anticoagulation therapy with rivaroxaban, the hemothorax was suspected to be associated with rivaroxaban use. Consequently, rivaroxaban was discontinued. Additionally, laboratory tests upon admission revealed abnormal coagulation parameters (Table 1), prompting the administration of 330 mL of fresh frozen plasma transfusion for management.

**Table 2:** Results of the pleural fluid by laboratory analysis.

Test item	Results
Color	Red
Appearance	Muddy
Red blood cells under high magnification	Full field of view
Red blood cells (× 10^9^/L)	1.433
Lymphocytes (× 10^9^/L)	0.30
Neutrophils (× 10^9^/L)	0.67
Macrophage (× 10^9^/L)	0.03
Total protein (g/L)	38.3
Albumin (g/L)	25.0
Globulin (g/L)	13.3
Adenosine deaminase (U/L)	19
Lactic dehydrogenase (U/L)	520
Glucose (mmol/L)	4.76
Amylase (U/L)	32
TB-RNA	Negative

TB-RNA, detection of tuberculosis ribonucleic acid.

**Figure 3:**
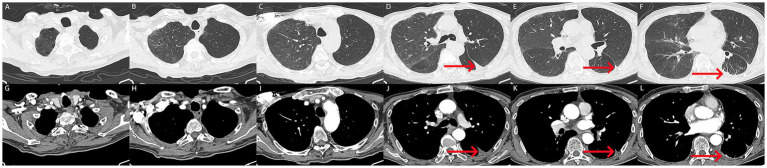
**(A–L)** After treatment, the patient underwent follow-up computed tomography imaging. **(A–L)** Computed tomography showed a marked reduction in hemothorax, with only a few pleural effusions in the left lung (red arrow). Atelectasis was improved, and no tumor related abnormalities were found in the lungs and pleura.

**Figure 4:**
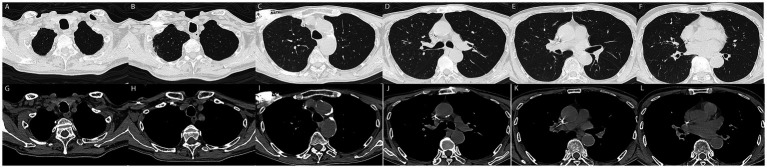
**(A–L)** 3 months later, the patient underwent follow-up computed tomography with no recurrent pleural effusion observed.

Following 5 days of treatment, the patient’s chest drain ceased to discharge hemorrhagic pleural effusion, and there was a significant improvement in the patient’s dyspnea, along with the normalization of coagulation function (Table 1). To rule out the possibility of pulmonary and pleural tumors, the patient subsequently underwent contrast-enhanced CT. The results indicated a marked reduction in hemothorax, improvement in atelectasis compared to previous findings, and no significant tumor-related manifestations in the lungs and pleura (Figures 3A–L). Consequently, the chest drain was removed, and the patient was discharged without complications (The timeline of key events is shown in Supplementary Figure S1). Fortunately, a follow-up CT scan 3 months later (November 12, 2024) revealed good re-expansion of the left lung and no recurrence of hemothorax (Figures 4A–L).

## Discussion

Atrial fibrillation, one of the most common clinical arrhythmias, has a high incidence among elderly patients, with a global prevalence of 2–3% (3). Due to the propensity for thrombus formation in the left atrial appendage during AF, the risk of ischemic stroke is significantly increased. Therefore, anticoagulation to prevent thrombus formation has become the primary strategy in the management of AF (4).

Warfarin, a vitamin K antagonist, is a commonly used anticoagulant in patients with AF. It remains widely used as a first-line treatment for AF, moderate to severe rheumatic mitral stenosis, or mechanical heart valve patients (5). However, with the advent of rivaroxaban (a direct oral anticoagulant), studies have demonstrated that rivaroxaban is either superior or non-inferior to warfarin in terms of anticoagulant efficacy. As a result, rivaroxaban has gained widespread acceptance in the treatment of non-valvular AF. Moreover, unlike warfarin, rivaroxaban does not require dietary restrictions or frequent monitoring of the international normalized ratio. Therefore, rivaroxaban is more highly recommended for patients with non-valvular AF (6).

Rivaroxaban is an oral factor Xa inhibitor with high bioavailability. It selectively blocks the active site of factor Xa and exerts its anticoagulant effect without the need for cofactors, such as antithrombin III. Rivaroxaban primarily inhibits the activation of factor X to factor Xa through both intrinsic and extrinsic pathways, thereby exerting its antithrombotic effects in the coagulation cascade (7, 8). Spontaneous hemothorax, defined as the accumulation of blood in the pleural cavity without any apparent trauma, is a rare clinical condition.

Research indicates that among individuals using rivaroxaban, the prolongation of PT and APTT is primarily associated with rivaroxaban plasma concentration and laboratory testing reagents (9). In this case, we hypothesize that the significant prolongation of PT and APTT may be related to high rivaroxaban plasma concentration, which further contributed to the occurrence of spontaneous hemothorax. Moreover, a retrospective study by Yuan categorized patients using rivaroxaban based on whether PT and APTT were prolonged. The analysis revealed that rivaroxaban could lead to mild prolongation of PT and APTT, but this mild prolongation was not associated with bleeding events. Additionally, the study indicated that height and hypoalbuminemia are independent risk factors for prolonged PT and APTT in patients taking rivaroxaban (7). However, due to the limitations of retrospective studies and sample size, further research is needed to confirm these findings.

Based on clinical experience and literature reports, conjunctival hemorrhage, gastrointestinal bleeding, genitourinary bleeding, gingival bleeding, subcutaneous hemorrhage, hemoptysis, and even intracranial hemorrhage have been reported during the use of rivaroxaban, with gastrointestinal bleeding and intracranial hemorrhage being the most frequently observed (1, 2, 10). However, spontaneous hemothorax is an exceedingly rare complication of rivaroxaban therapy. In this case, the occurrence of spontaneous hemothorax warrants further investigation. Studies suggest that concomitant use of nonsteroidal anti-inflammatory drugs (e.g., aspirin, ibuprofen, celecoxib, acetaminophen, etoricoxib), P2Y12 inhibitors (e.g., clopidogrel, ticagrelor), or fibrinolytic agents with rivaroxaban may lead to excessive drug exposure, thereby increasing the risk of bleeding events (11, 12). Additionally, low body weight, reduced creatinine clearance/glomerular filtration rate, and prior bleeding history are well-established high-risk factors for rivaroxaban-associated hemorrhage (2). Recent research has also highlighted the potential role of genetic factors in rivaroxaban-induced bleeding. A prospective multicenter study by Kim demonstrated that ABCG2 rs3114018 and ABCB1 rs1045642 gene polymorphisms are significantly associated with an increased risk of bleeding in patients taking rivaroxaban (13).

In this case, the patient had been taking rivaroxaban for AF for 2 years without any prior bleeding events. However, the medical history revealed that the patient had been diagnosed with esophageal candidiasis one and a half months earlier and received fluconazole therapy for nearly 2 weeks. Besides the oral administration of rivaroxaban and fluconazole, the patient was not taking any other medications, including aspirin, that could potentially cause coagulation abnormalities. According to the literature, approximately two-thirds of rivaroxaban is metabolized into inactive compounds by hepatic cytochrome P450 (CYP) enzymes, primarily CYP3A4 and secondarily CYP2J2, before being excreted in feces and urine (14, 15). In this patient, the overlapping use of rivaroxaban and fluconazole may have contributed to the bleeding event. Fluconazole, an azole antimycotic, is a known CYP3A4 inhibitor. Systemic administration of fluconazole likely suppressed CYP3A4 activity, leading to reduced rivaroxaban metabolism and subsequent drug accumulation, ultimately resulting in spontaneous hemothorax (16, 17). Studies have indicated that fluconazole, functioning as a moderate CYP3A4 inhibitor, also inhibits the transport protein P-glycoprotein to a certain extent. Systemic administration of fluconazole increases the exposure of rivaroxaban by more than 40%, thereby elevating the risk of bleeding (18). We performed a causality assessment using the Naranjo algorithm, with an overall score of 5 (Table 4). Therefore, fluconazole may have been the triggering factor for this bleeding complication. Unfortunately, the proposed causal relationship between fluconazole-associated elevation of rivaroxaban plasma concentrations and subsequent spontaneous hemothorax remains hypothetical in this case analysis, due to the unavailability of validated therapeutic drug monitoring (TDM) capabilities for rivaroxaban at our institution. This represents a critical methodological limitation of the present case analysis. When resources permit, we strongly advocate for the implementation of serial rivaroxaban plasma concentration monitoring during concomitant use with fluconazole to mitigate potential drug interaction risks.

In clinical pharmacotherapy, CYP3A4 inhibitors are not uncommon and warrant special attention when coadministered with rivaroxaban. Liu et al. (19) previously documented a case of rivaroxaban-induced spontaneous hemothorax in a 95-year-old male receiving long-term rivaroxaban therapy for deep vein thrombosis. The patient developed spontaneous hemothorax following itraconazole administration during hospitalization for pulmonary fungal infection. Similarly, our patient was on chronic rivaroxaban therapy for atrial fibrillation. However, distinct from their case, our patient developed spontaneous hemothorax after a 2-week course of fluconazole initiated for diagnosed esophageal candidiasis. Although different antifungal agents were implicated, both itraconazole and fluconazole constitute potent CYP3A4 inhibitors. Research has documented that spontaneous hemopericardium and cardiac tamponade may occur in patients receiving concurrent therapy with rivaroxaban and amiodarone (20–22). Notably, Elikowski et al. (23) further reported a case of hemoptysis associated with the concomitant use of rivaroxaban and CYP3A4 inhibitors, such as amiodarone. Table 3 summarizes the clinical profiles of these cases.

**Table 3:** Clinical profiles of patients receiving rivaroxaban interacting with CYP3A4 inhibitors.

Authors	Country	Year of publication	Study design	Case number	Age/sex	Main symptoms	Drugs interactions	Diagnose
Liu et al. (19)	England	2021	Case report	1	95/male	Dyspnea	Rivaroxaban/itraconazole/caspofungin/bisoprolol fumarate/isosorbide mononitrate/spironolactone/tiotropium bromide/salmeterol fluticasone/ambroxol hydrochloride/ferrous succinate/vitamin C/esomeprazole	Spontaneous hemothorax/pulmonary fungal infection/chronic obstructive pulmonary disease/hypertension/hiatal hernia/deep vein thrombosis
Lefas et al. (20)	UK	2020	Case report	1	86/male	Breathlessness and bilateral leg swelling	Rivaroxaban/amiodarone	Spontaneous haemopericardium and acute kidney injury
Oladiran et al. (21)	USA	2018	Case report	1	87/male	Chest pain and light headedness and shortness of breath	Rivaroxaban/amiodarone	Spontaneous hemopericardium and cardiac tamponade
Menendez et al. (22)	USA	2016	Case report	1	69/male	Palpitations and chest discomfort	Rivaroxaban/atorvastatin/dronedarone/omeprazole/tamsulosin/tadalafil/cyclobenzaprine	Hemopericardium
Elikowski et al. (23)	Poland	2015	Case report	1	75/male	Hemoptysis	Rivaroxaban/amiodarone	Unilateral pulmonary with ground-glass opacities

**Table 4:** Naranjo algorithm—ADR (adverse drug reaction) probability scale.

Question	Yes	No	Do not know
1. Are there previous conclusion reports on this reaction?	1		
2. Did the adverse event appear after the suspect drug was administered?	2		
3. Did the AR improve when the drug was discontinued or a specific antagonist was administered?	1		
4. Did the AR reappear when drug was readministered?			0
5. Are there alternate causes [other than the drug] that could solely have caused the reaction?			0
6. Did the reaction reappear when a placebo was given?			0
7. Was the drug detected in the blood [or other fluids] in a concentration known to be toxic?			0
8. Was the reaction more severe when the dose was increased or less severe when the dose was decreased?			0
9. Did the patient have a similar reaction to the same or similar drugs in any previous exposure?			0
10. Was the adverse event confirmed by objective evidence?	1		
Total score = 5			

Score ≥9 = definitive, 5–8 = probable, 1–4 = possible, ≤0 = doubtful.

Furthermore, spontaneous hemothorax associated with rivaroxaban monotherapy has been previously documented. For instance, Al Hariri et al. (24) presented a case of a 49-year-old male who developed spontaneous hemothorax while receiving rivaroxaban for pulmonary embolism. Additionally, prior reports suggest that advanced age (>65 years), reduced creatinine clearance, low hemoglobin levels, drug overdose, and hepatic impairment (manifested as coagulopathy) may represent potential risk factors for spontaneous hemothorax in patients undergoing rivaroxaban therapy (19, 25, 26).

As rivaroxaban-induced bleeding may occur in various anatomical sites, individualized treatment and management strategies should be implemented based on the severity and location of bleeding. In general, for patients receiving rivaroxaban who experience minor or localized bleeding, temporary discontinuation or delayed administration of the drug should be considered as the first step. However, immediate cessation of rivaroxaban is crucial in cases of major bleeding. In this case, the patient presented with spontaneous hemothorax accompanied by dyspnea. Upon admission, the patient received oxygen therapy and underwent therapeutic thoracentesis, and placement of a chest drain. Furthermore, laboratory tests upon admission revealed significant coagulation abnormalities, prompting administration of fresh frozen plasma to the patient. Notably, administration of prothrombin complex concentrate (PCC) or fresh frozen plasma (FFP) should be considered as an important therapeutic option in patients receiving rivaroxaban who develop severe persistent bleeding accompanied by coagulopathy (27, 28).

In addition to spontaneous hemothorax, gastrointestinal bleeding and intracranial hemorrhage are more frequently encountered in clinical practice. For patients with severe gastrointestinal bleeding, activated charcoal administration may reduce rivaroxaban absorption in the gastrointestinal tract (29). Furthermore, antifibrinolytic agents (such as tranexamic acid and aminocaproic acid), as well as prothrombin complex concentrate (PCC), activated prothrombin complex concentrate (aPCC), 4-factor prothrombin complex concentrate (4F-PCC), or recombinant activated factor VII play crucial roles in managing rivaroxaban-associated bleeding (28, 30). Regarding life-threatening intracranial hemorrhage, studies suggest that in addition to immediate rivaroxaban discontinuation, patients should receive factor Xa reversal agents (such as andexanet alfa) (31, 32). Surgical interventions, including hematoma evacuation by neurosurgeons, may also be required when necessary.

This case underscores the critical importance of vigilance for hemorrhagic events when rivaroxaban is administered concomitantly with other medications, particularly CYP3A4 inhibitors. Although spontaneous hemothorax represents an exceedingly rare clinical entity, its possibility should be considered in rivaroxaban-treated patients presenting with dyspnea and radiographic evidence of pleural effusion. Furthermore, close clinical monitoring for symptoms is warranted during co-administration of rivaroxaban with CYP3A4 inhibitors. At institutions where feasible, monitoring anti-Xa levels may provide an additional strategy for hemorrhage prevention.

There are important strengths and limitations of this study to consider. Strengths include the patient’s advanced age and male sex being offset by his limited comorbidity burden and simple long-term medication profile, which greatly facilitated our analysis of spontaneous hemothorax pathogenesis. His hospitalization at our center ensured comprehensive collection of laboratory parameters, and radiographic data. High treatment compliance further contributed to the favorable clinical outcome.

Limitations include the outpatient administration of fluconazole, which rendered potential confounding factors influencing spontaneous hemothorax development during unsupervised dosing unassessable. Compared to conventional coagulation tests (including INR and APTT), anti-Xa activity measurement serves as a more reliable indicator of rivaroxaban exposure *in vivo*. The unavailability of rivaroxaban-specific anti-Xa assays at our center constitutes an important methodological constraint.

## Patients’ perspective

The patient reported significant distress due to progressive dyspnea over 1 month, severely limiting his daily activities and mobility. He expressed primary concerns regarding the bloody pleural fluid observed during chest drainage and anxiety about potential underlying malignancies given his age. Following detailed counseling by the clinical team, he acknowledged understanding the suspected drug interaction between rivaroxaban and fluconazole as the likely cause. He actively complied with rivaroxaban discontinuation and transfusion therapy, noting marked alleviation of dyspnea within days post-treatment. At follow-up, he conveyed gratitude for the resolution of symptoms and heightened awareness of medication-related risks in future care.

## Conclusion

This case highlights the potential risk of spontaneous hemothorax in patients concurrently receiving rivaroxaban and fluconazole. Furthermore, it underscores the importance for clinicians to be vigilant about drug–drug interactions when formulating medication regimens. A thorough understanding of the patient’s medical history is essential. For high-risk populations using rivaroxaban who are prone to bleeding, more intensive monitoring is necessary, including dynamic monitoring of anti-Xa factor activity when required. For patients on multiple medications, regular outpatient follow-up and monitoring should be implemented to promptly identify and address any related clinical issues.

## Data Availability

The original contributions presented in the study are included in the article/Supplementary material, further inquiries can be directed to the corresponding author.

## Supplementary material

SUPPLEMENTARY FIGURE S1 The timeline of key events.
