# Supplementary figures and images for: Rivaroxaban-induced spontaneous hemothorax: a rare case report and literature review
Source: Front Med (Lausanne). 2025 Jul 29;12:1641092. doi: 10.3389/fmed.2025.1641092 (PMC12339356; doi:10.3389/fmed.2025.1641092)

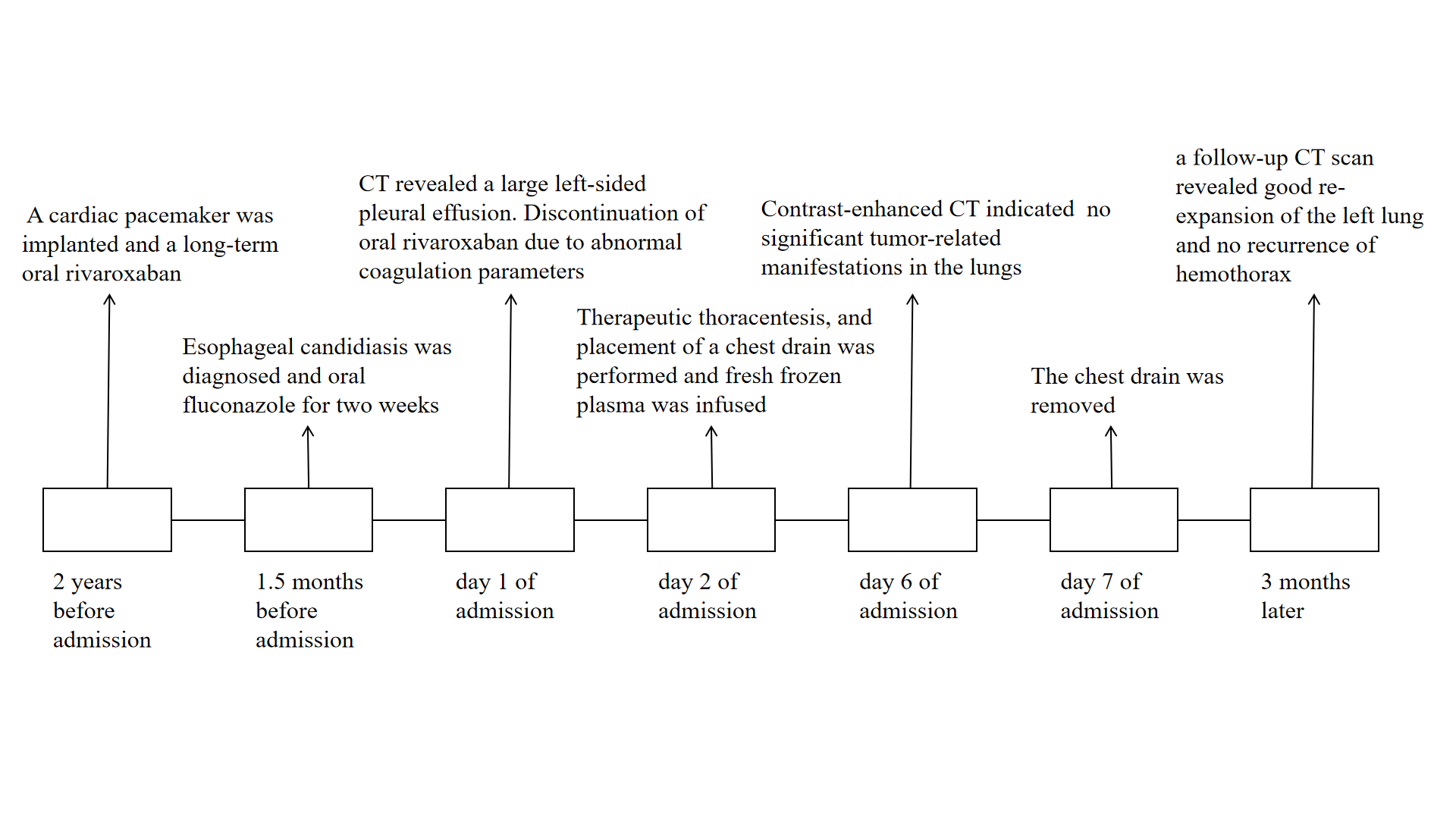

Supplement: SUPPLEMENTARY FIGURE S1 — The timeline of key events. [file Image_1.tif]
